# Union Meeting of the Connecticut Valley Dental Society, the New England Dental Society, and the Connecticut State Dental Association, at Springfield, Mass., October 23, 24, and 25, 1889

**Published:** 1890-03

**Authors:** 


### UNION MEETING OF THE CONNECTICUT VALLEY DENTAL SOCIETY, THE NEW ENGLAND DENTAL SOCIETY, AND THE CONNECTICUT STATE DENTAL ASSOCIATION, AT SPRINGFIELD, MASS., OCTOBER 23, 24, AND 25, 1889.[1]

THE meeting was called to order at 2.30 P.M. by Dr. E. S. Gaylord, chairman Executive Committee.

*Dr. Gaylord.*—Gentleman, the hour has arrived for calling this meeting to order. I now have the pleasure of introducing the president of the Connecticut Valley Dental Society, Dr. S. B. Bartholomew.

### DR. BARTHOLOMEW'S ADDRESS OF WELCOME.

GENTLEMEN AND PROFESSIONAL BRETHREN,—Through the courtesy extended by your Executive Committee I have the honor, as well as the great privilege, of welcoming, from all parts of New England and the Canadas, the members of the Connecticut Valley, the New England, and Connecticut State Dental Associations, together with our invited guests from different sections of the country, to the autumnal beauties of Springfield as well as to this dental gathering.

Our good mayor, a few weeks ago, placed a pot of red paint in the hands of the members of a section of the Grand Army of the Republic, who chanced to hold their annual reunion in this city. He bade them enjoy themselves to the utmost. Although I cannot speak with municipal authority, I will say this much, that, if you find less leniency shown you than was shown on that occasion, woe be to any one of our present municipal authorities who shall hereafter demand the professional services of any of the local dental committee who has assisted in completing the arrangements for this convention, for he will proceed to square the account by inflicting the full pains and penalties known to dental science.

It affords me great pleasure to stand in this place as a sort of link between the past and the brilliant future that is before us, and open the door to the work of this dental convention. I am now in my fifth dental decade. It covers a period of the world's his-

---

[1] Reported for the INTERNATIONAL DENTAL JOURNAL by Geo. A. Maxfield, D.D.S., Holyoke, Mass.

tory in which the arts of peace have attained their greatest triumphs. The impartial historian will place the art and science of dentistry as *one* among the new births or revelations to man's consciousness during this period, out of which is coming the greatest possible good to mankind. Little by little we have seen the flame of incense ascending from our dental altar, representing the full vigor of early manhood, the science of investigation, the restless genius of invention, the spirit of refinement and culture, until the whole world pays tribute to American dentistry. And in this convention I am proud to recognize men who have accomplished their proportional share in this great work. In the fall of 1848 I entered, as a student, one of the best dental offices in Massachusetts. At that time, I believe, there were but four dental graduates in all New England. We were like travellers in a wilderness, with here and there a blazed tree to guide us. It was fortunate for us that there was then no law against any man's experimenting on the dear public to his heart's content. But, notwithstanding all that has been done to reduce dentistry to an art and a science, it has as yet no clearly recognized place in the field of M.D.ism, and will not until the curriculum of study shall be such as to place the graduate in dental medicine and dental surgery upon an equality with the graduate in any department of medical and surgical science.

The tendency is towards the highest possible attainment in all professional life, and the man who stops at the attainment of his D.D.S. will find himself, ere long, barred against associating on terms of equality with the constantly increasing number of M.D., D.D.S.'s.

A calling that would attain to the grand and the great rises above the every-day grad-grind for subsistence, and carries its clan into the realms where work is transformed into the intensest pleasure under the stimulus that comes from the pursuit of the unattained, and draws towards it a constantly ascending grade of mental force and power. Then we may look for a realization, in the near future, of the dream of our advanced brethren who are bearing the heat and burthen of the day, not the dream of Longfellow's alpine youth, whose burthen of strange desire was lost to sight and realization, as he seemed transformed into a lunatic above the clouds, leaving behind him his mortal vehicle stuck fast in snow and ice; not the utopian dream of Sir Thomas More, whose ideal was beyond mortal attainment, but the dream of an educated and scientific art that shall move by the side of and be classed with the learned professions. I expect to live to see the day when the Harvard school will, in obedience to a demand, require the M.D. as a foundation upon which to lay the D.M.D.

Gentlemen, may your stay in our city be agreeable to you, and may the sessions of this convention be profitable to us all.

*Dr. Gaylord.*—Gentleman, Dr. C. A. Brackett, president of the New England Dental Society.

Mr. President and Members of this Convention,—It gives me pleasure to appear before you and utter a few words in the capacity in which I am permitted to speak. As it was my privilege to say on a former occasion, the occasion of my accession to the presidential position which you now so capably fill, the Connecticut Valley Dental Society is like a home to me. It was the first dental organization to enroll me as a member. Among the members of this society are men who have been to me most helpful as a student of dentistry. From the men whom I now see before me, I think I have gained, in a sense, more courage and help than from any men in the world. So it was a pleasure to come back to its society meetings. It has not been convenient or possible for me to be a regular attendant at the meeting of late years, and I come back as the "prodigal son," not as one having engaged in riotous living,—for dieting on clams is not to be called riotous living,—but I come back in the sense of the prodigal son who has been away for a time from his father's house. If you will kindly let me indulge in a few personalities, I will speak of the way in which I was impressed on my arrival in your city after dark. There was a sense of bewilderment, due to the change of the railroad station. I needed guidance and direction to get out on the main street. Since my last visit, great changes, I find, have taken place; new hotels, new stores, the enlargement of buildings, etc., and enterprise, vigor, and energy appear on every hand. In the morning I rose early and went eight miles to Holyoke, which, only a few years ago, had a population of but ten thousand, and now has over thirty-three thousand, and with positive evidence of life and energy or push and progress that, to a person who has been living in a place where nothing is done in business, was inspiring. The Connecticut River I studied about in school, as a matter of geography, rises in Connecticut Lake, and flows south through Massachusetts and Connecticut, and empties into Long Island Sound. The Connecticut River is a great institution in itself, yet, compared to the Connecticut Valley, is a small thing. The Connecticut Valley, with the accompaniment of the Connecticut Valley Dental Society, is a thing of wide extent. We might then bring in a little more geography, and say, the Connecticut Valley is bounded on the north by Montreal; and speaking of this, it reminds me that the society is always glad that it has such a good, big "Bazin." The Connecticut Valley is bounded on the east by Uncle "Dudley," and he spreads over a good deal of territory himself, and will doubtless do so to-day. On the south, by salt water,—let us hope it will never be "Salt River." On the west, by the setting sun. We see no indications that the sun ever will set in this valley. The Connecticut Valley has large dimensions in height, especially here in Springfield, and has always been so. In Boston we have deep water, that, whatever the "Weatherby," we are not likely to have its "Niles" "Leach" away; but here in Springfield you go below water into profound "Mayr." Yet let any man "Hurlbut" a bottle at the fair fame of your society, and we shall all rise in its defence. It is a society we know full well, for you always keep its "Stockwell" up to par. When it comes to largeness of heart and helpfulness of hand, I tell you, Mack, there is no one who can beat "McManus." The Connecticut Valley Dental Society, in times past, has reached some new ideas, and they are ideas that are not likely to become "Snow"d under. Its members have in their time run against a good many snags, and still succeeded in living, but whenever we go to Brattleboro', we are always glad to run against a "Post." The old saying, "it takes nine tailors to make a man," we have heard, but it is demonstrated to us that when it comes to a matter of working on committees or to engineer a steamboat excursion, one "Taylor" is equal to nine ordinary men. Our societies are, in a sense, cosmopolitan. New York can match our "Smith" and "Jones" with their "Robinson."

In all the push and enterprise in Holyoke, I could not help thinking, with all its "Hastings," it is building solidly, and with all its material with which to build, it needs to be to complete the culture of its fields, and the most satisfactory one to meet is its "Maxfield." You know the reason given for giving up the reading of the Bible in the public schools of Minnesota was because it made so much more mention of St. Paul than of the other cities; yet I have never heard any one make any opposition to St. "Bartholomew." Our bishop may be absent, but our "Shepard" is always here. If we could only have our friends from Buffalo with us, we would try to "Barrett." I should not, however, forget that we came here for something else than such things as we have been speaking about, and I wish to make mention of how much we owe to this society, the Connecticut Valley, and the New England Dental Society, for which latter it is my honorable privilege to speak. Seriously, a very large part of the progress in our art and science that has been made in New England has been accomplished through the pioneers who have kept up their active work in both societies, and have greatly contributed to the advancement of the cause in which we labor. Notwithstanding the time and manner of our calling, a very large proportion of the members of these societies are in good health and in the active pursuit of their calling. I was noticing just before leaving home—and this may be stated as a fact—that the dentists are, as a class, men of good health that bid fair to attain longevity, and are a model of perfect development.

We are very glad to be with you on all occasions, and if we do not come, it is because we are creatures who cannot control all our ways. I thank you for the courtesy of the introduction and for your listening to me. I look for much profit from these sessions in the days to come.

*Dr. Gaylord.*—Gentlemen, Dr. W. J. Rider, president of the Connecticut State Dental Association.

RESPONSE OF PRESIDENT RIDER.

Mr. President and Gentlemen of the Connecticut Valley and New England Dental Societies,—It is my privilege, in behalf of the Connecticut State Society, to thank you for the warm welcome we have received from you, to express the pleasure we have of meeting with our professional brethren, and to discuss some of the great questions pertaining to our specialty. I say brethren, for we are brothers working together to do what we can to ameliorate some of the conditions of life in our fellow-men. And we may congratulate ourselves that the day has long gone by, never to return, when the secrets of the laboratory and the operating-room never went outside the office-door; when what a man knew he kept to himself, jealously guarding from his brother practitioner his own mode of operating. The opposite of this is the rule now, for as soon as a man arrives at any new mode of treating a case, he at once, by a paper read before his society or by an article in some one of the many dental magazines of the day, gives to the profession the results of his researches,—anxious to add his brick to the pile of knowledge.

And this leads me to say that dentistry is fast becoming a liberal profession; no longer narrow or contracted, but bestowing with a free hand the exertions of the mind, as well as the results of the labor of the hands.

I envy the young men coming into practice now; they are starting in life where the older ones are leaving off; they have the benefit of all their researches and experiments; the accumulated knowledge of the past is at their command, and they have but to go on to still higher attainments. They little know the difficulties and discouragements that the student had to overcome in the days when your speaker commenced practising. In the larger cities were some educated men, but the mass of the profession throughout the country was very ignorant. We had but few works on dentistry. I think but two dental journals were published in the country, and they were very crude. No dental societies; and everything that a man knew he kept to himself, so that there was no general dissemination of knowledge on the subject.

And this reminds me of an incident that occurred, one day, while pursuing my studies. I was reading Harris's "Principles and Practice of Dentistry," when I came across the word periosteum. Says I, "Doc., what's the periosteum?" "It's aw—aw—it's; well, it's—you know if you drive a nail in a board, it stands in the board so?" "Yes." "Well, that's it." That was just as lucid as mud. Of course, I knew all about the periosteum and its office in connection with the teeth after that explanation. I am happy to say that that gentleman has kept up with the growth of dentistry. He has enjoyed a large practice in the city of Brooklyn; he had the honor of calling to order the first meeting called to organize the Odontological Society; and was at one time a member of the board of censors that meets at Albany to examine candidates for the title of M.D.S.

The higher ethics of all liberal professions require that each member shall give in full the results of his investigations for the benefit of the profession at large. It condemns the man that takes out a patent on any new method of operating, or instrument, or sells to any one instrument-maker the right to make and vend the same, thereby depriving the profession of the full right to make and use them. It requires that the little that any one man may learn in a lifetime should be given freely in return for the great stock of knowledge he is at full liberty to draw from. Or, as the good book puts it, "Freely ye have received, freely give." When every member of the profession learns this, and lives up to it, then shall we be truly—and not until then—what we claim to be, a liberal profession. Those of us who paid tribute so long to the rubber company, and fear the rod that the tooth company holds over us, feel the force of what I say.

Mr. Chairman, the future of dentistry looks very bright. Looking back over a practice of forty years, I am impressed with the strides that it has taken.

The advent of the rubber dam, the dental engine, the mallet in its various forms, cohesive gold, and the many other appliances of the day, have revolutionized our methods of operating, and enabled us to arrive at results that the fathers never dreamed of. Reviewing what has been done in the last twenty-five years, who dares prophesy what are the possibilities of dentistry in the next quarter of a century, or what will be the modes of operating? We may congratulate ourselves that there is so much working together for good for the advancement of our profession,—the many dental colleges, where, under competent instructors, a young man may get a good education; the many periodicals, filled with valuable papers, and discussions upon every new thought and thing; the many dental societies, where our best men get together to compare results; and last, but not least, the laws regulating the practice of dentistry in the different States, keeping out the quacks and illiterates, requiring a higher standard of education in the practitioner, —thereby protecting the profession, and insuring to the public a better class of operators. All these things are tending towards a higher standard in the future, and are hastening the day when oral surgery will be fully recognized as a specialty of the healing art.

Thanking you for the cordial welcome we have received, I close with the wish that the auspices of the present moment may continue, insuring the success of this our first union meeting in order that it may be an enjoyable one, and of great profit to all.

Dr. W. C. Barrett, of Buffalo, being called upon, responded as follows:

I can only say it affords me great pleasure to meet with you. It is not the first time, and I hope it is not the last. I shall not attempt to imitate the purpose of my friend who was speaking when I entered the hall, for you know little sayings are always put in "Bracketts." I will only say this, I have come once more to meet with you here, and I only hope you will receive as much benefit from me as I shall from you. I know I shall carry away from here a great deal more than I bring. I have always carried away my head full and sometimes my pockets. It affords me pleasure to meet these friends and to attend these meetings, which have always been profitable, and I am sure will be now.

Addresses closed.

*President Bartholomew.*—We will now proceed with the regular order of the programme. The first on the list is a paper by Dr. E. S. Niles, of Boston, on "The Inference of Adenoid Growths in Oral Deformation."

*President Bartholomew.*—Gentlemen, the paper just read is now before you for discussion.

*Dr. Porter.*—Are we to understand that these specimens shown came from a single mouth or from a number of mouths?

*Dr. Niles.*—From a number of mouths. They are specimens borrowed from Dr. Hooper. Each bottle contains what was removed from one mouth.

*Dr. Morgan.*—Do you consider the high-arched mouth or palate the cause of this condition, or is this condition the result of a high-arched mouth?

*Dr. Niles.*—Of course the stoppage of the passage through the nose, causing mouth breaking, exerts an influence on the palate.

*Dr. C. A. Brackett.*—This subject, which Dr. Niles has brought before us to-day, I have been led to give some attention to, through a previous effort of dentistry in another place. It has seemed to me a matter of very considerable consequence. My own idea about it—and I make no pretence as to correctness—is that the varying of the upper arch at the sides and the bulging up of the hard palate is in consequence of the deviation of the muscles, through keeping the mouth open. It seems to me, with the very limited attention given to the subject, that I should endeavor to stop this difficulty. As, for instance, in a case of a child breathing with difficulty or noisily, especially at night. In some of these cases that have been operated on he makes mention of very great alarm being excited because the child slept with perfect quietness after the operation. The child was a noisy sleeper, and immediately upon removal of these growths the breathing became so quiet that there was anxiety on the part of the parents, thinking it of some grave consequence. Dr. Hooper says he removes many of these growths with his finger-nail. I think that a dentist is as capable as any person to make the examination. If a case came to me, I should feel justified in making the examination, and under the influence of nitrous oxide, if the age of the patient made it advisable. I cannot see any harm in making the examination. If I did not feel like doing that, I should refer the patient to some one who had had practical experience with the treatment of this affection.

*Dr. Maxfield.*—Since reading the paper of Dr. Hooper, referred to by Dr. Niles, which I first saw a few months ago, I have been very much interested in this subject. One thing that Dr. Niles did not mention was that Dr. Hooper says that it was in 1885 that he first removed these growths, and thinks he was the first one to perform this operation in New England. I refer to this because it shows that there is not much known about this affection in the medical profession. A few weeks ago a child was brought to me by her parents to have its teeth straightened. As the child had many of the characteristics that Dr. Hooper attributes to this affection, I advised the parents to have a specialist make an examination of the throat and nose. They did so, and reported that the examination had been a very thorough one, and that nothing of the nature I had alluded to had been discovered, and that the specialist attributed the mouth-breathing to catarrh. I asked how the examination had been made, and they answered, by passing an instrument into the front of the nose. While this examination satisfied the parents, it was far from being satisfactory to me.

*Dr. L. D. Shepard.*—I have seen a considerable number of the patients that have been spoken of, and quite a number of my patients have been operated upon by Dr. Hooper. I have no question, from what little thought I have given to the subject, that the collapse of the upper jaw is caused or helped by this growth. I have not given any special study to this subject in a proper scientific manner, but almost all the patients with whom I have been familiar, that had this trouble, have also had accompanying it the peculiar conditions of the mouth already spoken of. The essayist did not speak of what has always seemed to me a very interesting circumstance connected with the operation which we have the impression of as being original with Dr. Hooper. You well know, in all bloody operations in the mouth, the difficulty occasioned by the blood passing down the throat into the œsophagus. Dr. Hooper in these operations places the patient prone upon the face, so that the blood comes out of the mouth. All patients, young or old, when etherized, are placed upon the table, face down, head hanging over, and the operation performed by sense of touch ; there is no trouble from blood as it drips out into a basin. This has solved with him the whole difficulty of giving ether with a bloody operation in the mouth. I think this subject a very interesting one, and am glad it has been brought before this meeting. The evidence of this condition is most marked in the patient, and you can recognize the patient the moment you put eyes upon her or him. I have in that way noticed several of my patients, and have sent them to Dr. Hooper, and my diagnosis has been confirmed by him. We all know that mouth-breathing and irregularities of the teeth go together, and we have both in this disease. It is of so recent discovery, it has escaped notice until now. Another feature, and a very prominent fact, in regard to this subject is, it is a self-limited disease. As a rule, it is a disease of youth and not of adult age; my impression is it is very rare to find such a case in an adult.

*Dr. Wm. H. Atkinson.*—It is useless for me to improvise and talk of principles and facts that we have not been made acquainted with. There is knowledge on this subject which acts without saying. Most of the remarks that have been made are simply surmises. The man who handles the mouth must and will be an expert at it. As to the cause of this trouble we know absolutely nothing. We know indications, and yet every indication, as noted, we do not know, for we cannot pursue it beyond the reach of mental grasp. These pathological growths cannot be because of food formation, but must be from the need of nutrition of the part; they are evidently hard and epithelial-like; the myxomatous matter of the gum is here involved outside of its immediate condition. There is a need of considerably enlarged vessels here, and until these vessels have been enlarged the growths continue. We know so little of it that I only care to emphasize our ignorance, and call for a ratiocination of our voice and mind until, with the inspiration of the moment to guide us, we can retain what we know; until then it will be useless to talk about it. One remarkable case I wish to speak of, where I have made fourteen or fifteen operations to remove the abnormal growths which filled the entire buccal cavity and covered the cheek in folds like the pouches of a chipmunk. If I ever get my saddle-legs on, I will write this case up; it is of too much importance and interest to let go. It was entirely of epithelial growth. I applied a solution of salicylic acid in alcohol, and just cooked the abnormal growth, and was thus enabled to remove it. It had not involved the blood tracts. My place is so much in the possession of my dental friends that I am not always able to put my hands on the specimens I wish to, so was unable to bring them with me. We have not enough to indicate what this pathological growth is.

What this term adenoid means we do not know. It is in this mixture of terms where we shall get into difficulty, and if we are not careful we shall get into greater difficulties from them. We shall be pushed from one supposition to another, until at last we hope to take such a grip that it will be understood by all.

*Dr. Shepard.*—I think the essayist gave the definition of adenoid, and with sufficient emphasis for all to recognize it. It is a mere name and means gland-like, which, however, does not fully describe it.

*Dr. Atkinson.*—It is a pouch in contact with the tissue, is lined with epithelial cells, and depends upon them for activity.

*Dr. Niles.*—As I understand it, this adenoid growth belongs to the glandular growths of the throat, and they are located in the region of the pharynx. They are simply a hypertrophy of the cells of the pharynx. Dr. Hooper says the old fashioned polypus is not the same as the adenoid growth; but, of course, a man who has a specialty of this kind is liable to go to extremes, the same as in other specialties. A young man of my acquaintance says his grandfather died of polypus. This young man goes about with his mouth open, and respiration would be entirely shut out if it were not for the mouth. His parents are very much prejudiced against having anything done. Another case: a gentleman from Chicago came on to me with his two boys to have their teeth straightened. These boys have these adenoid growths, and their father intended to have an operation performed. I succeeded in getting a very good arch, but their parents then refused to have anything done. I was well convinced that nothing permanent would result till this operation was performed. Dr. Young treated them with escharotics. Most of the information we have is mainly from Dr. Hooper, though Dr. Bradbury has made some study of the trouble, and one of the specimens that are being passed around was taken from the mouth of one of Dr. Bradbury's children. I myself have four children which I am going to have examined very soon. I believe they have this growth; they all show indications of it, having the hacking cough. The youngest is about one year old.

Subject passed.

### NEW APPLIANCES AND DISCUSSIONS.

*Dr. N. Morgan.*—I have here a little broach-holder I made for my own use. I was so much troubled with my broaches slipping. This is made from one of the cone-socket instruments,—an excavator. It was screwed into the handle and cut off within one-fourth inch of the handle, then taken out and a hole drilled through, the size of the broach; next, with a saw a slot was cut half the length of the hole. As you screw it into the handle it squeezes the sides together, thus holding the broach very firmly. I also have here some exploring points that are made from piano wire, simply filed down and bent into the shapes desired. These bone handles are simply needles—such as ladies use for knitting worsteds—cut in two and holes drilled in them, and the wire is cemented in with shellac. These needles can be obtained at any fancy-goods store, and only cost from fifteen to twenty cents a pair,—enough to make four handles.

*Dr. J. F. Adams.*—I have here a modification of the McLean disk; it is simply a metal disk soldered to the mandrel instead of fastening it on with a screw. This disk is made of brass, and, after it is shaped to run true, the emery paper is glued on, using a solution of dextrine. When the paper is worn out, it is dipped into a glass of water, when the paper will come off. The advantage of not using a screw is that you can use them for sharpening knives.

I also have a syringe here that is home-made. A short time ago I was treating a case of pyorrhœa alveolaris, and I wanted the patient to use a syringe at home to wash out the pockets. I went to a rubber-store and bought a small rubber ball that cost three cents, then took a glass tube from a medicine dropper, inserted it into the ball after enlarging the air-hole, and you see I have a perfect syringe.

I also have here a method for removing roots of teeth. The idea is not original with me, but there are some here who perhaps have never used it. I had a patient where the roots of the teeth that were broken were more or less covered with the gum, which was very sensitive. I took the engine and drilled into each root, then put in a wood screw, and removed all the roots without touching the gum.

*Dr. E. A. Stebbins.*—In taking impressions, especially of upper cases, I always found, when putting the finger up to hold the cup, that the cup would slip forward. To obviate this, I have had a piece of tin soldered on to the under part of the cup so that my finger rests against it, and the tendency is to press the cup back all the time.

*Dr. L. C. Taylor.*—A few weeks ago, looking around, I came across a practical little thing for holding the rubber dam, instead of using weights, which are very tiresome to the patient when sitting for a long operation. This holder is made of piano wire bent in the shape of a bow. On the ends are fastened large beads.

A weight is fastened to the bow. The dam is stretched over the ends of the bow, and the spring of the bow keeps the dam stretched out of the way. If small balls are not used on the ends, they will punch a hole through the dam. This is the invention of a Hartford man, and has been placed on the market, and sells for fifty cents.

*Dr. E. S. Gaylord.*—What I have to present is a little device I have enjoyed using very much. One is a rubber dam clamp and separator combined, the invention of Dr. Clough, of Boston. Another thing I find serviceable in the laboratory or elsewhere is an appliance in the form of a bench-block. It is not my invention, but I have used it several years. It is simply a cork. A hole is bored in the block and the cork is glued in. It is valuable for filing a screw or small article, as they embed themselves into the cork. I have here a band that I have had manufactured for my own use. I use the Bonwill engine. This band is a hard-finished linen thread band and has a core; this does not have any lint, which those who use the Bonwill engine will appreciate. All those of my friends to whom I have sent this band endorse it. Some weeks since, my friend here, Dr. Maxfield, instructed me how to make a splice, and until then I had been greatly troubled, for there is a constant breaking of bands, and the old method of splicing, which must be done with needle and thread, consumed so much time and was so troublesome to keep in order. So, not to be detained when a band broke, I have kept two engines. This now is not necessary since Dr. Maxfield has given me this splice, for there will be no more such trouble with splicing bands. It is a perfect success. I have tried to get the S. S. White Company to put this band on the market, but as yet they have not done anything about it.

*Dr. G. F. Harwood.*—I have here a vertical jack. All of you, doubtless, know what a jack is. This is made for carrying emery wheels, which will enable you to grind with the wheel horizontal. You can grind either on the top of the wheel or on the edge. These emery wheels are made in Worcester, and you can get them of any diameter. The diameter of this spindle varies about two-hundredths of an inch, so that the wear can be taken up by the screw which it rests upon at the bottom. This jack can be placed on the front edge of the bench and can be connected to a motor or a foot-wheel. I have also drilled a hole in the top of the spindle so as to carry a mandrel such as we use in the engine, and so run the McLean disks. You can do your rough grinding on the wheel and finish on the disks. The emery wheels are made solid with a soft metal centre so as to fit your mandrels. They come in varying grades, both as to hardness and fineness, running from No. 80 to 150. These grades, which are coarse and soft, are best designed for quick cutting. An instrument, after it has been tempered, should be sharpened on a wheel that is soft, such as will readily give way to the instrument. You, of course, want a coarse one to start with and a fine one to finish with.

*Dr. J. K. Wiley.*—I have here what are called plate screws, and I use them for regulating, for drawing in and pushing out the teeth. They are steel screws, and can be obtained at any watch factory or of any jeweller. They do the work for me better than anything I have ever used.

*Dr. Gaylord.*—I had the pleasure of seeing, the other day, something which seems to me to be very promising to future new engines. It is the invention of Dr. Campbell, of New York. You will remember him as the dentist who used to do celluloid work. This is an engine which has a horizontal driving-wheel. It can be placed under your chair out of sight. The foot-piece can be moved around anywhere,—if the legs of the chair are not in the way,—into any position you may wish. The rod comes up back of the chair, and you can attach any of the arms you may wish to it. It works very beautifully. The principle of the engine is the same as in the Bonwill, and you can get the same power and speed. Another very nice feature is that you can fold it up and put it in a case, the size of a violin case, and carry it anywhere. Dr. Campbell will soon put the engine on the market. I think we have something in this new engine which will be a great improvement over any engine now in use. It is out of the way and runs very smoothly. One of the English dentists, to whom Dr. Campbell had loaned this engine for a month for him to experiment with, declined to give it up when Dr. Campbell sent for it, and sent him instead his check for two hundred and fifty dollars. The appreciation of those who have seen it is very great, they are all very enthusiastic about it. It is a valuable acquisition, which we shall probably have, provided it gets into the proper hands.

*Dr. W. C. Barrett.*—I want to say a few words, and it is simply this, the dentist who attempts to stand on one leg and run an engine with the other is going to be at a disadvantage. For a number of years I have had the most simple contrivance and it has given me more satisfaction than anything else I have ever had. It is a water motor. It is one that does not require more than ten pounds pressure to run. The motor I have I can cover with my hat. It can be placed beside the chair and makes no noise. I have used it for a good many years. The cord from the small pulley of the engine is carried down and around the chair to the water motor. The valve of course is controlled by the foot, this turns the water on and off; just the slightest amount of pressure by the foot gives more or less speed, and it can be stopped instantly. My advice is to get a water motor and be relieved from all the trouble you ever had in regard to the running of the dental engine.

DISCUSSION.—DENTAL PROTECTIVE ASSOCIATION.

*Dr. Shepard.*—My appointment to open the discussion on this subject was made without consulting me, but I will try to discharge the duty. All the dentists here present have received one or more of the circulars of the Dental Protective Association. The majority have doubtless laid the circulars to one side without reading them. Some have responded favorably, and others have said to themselves that they would do so at a convenient (future) time. Both those who have not read the circulars and those who have not already joined the Association have made a great mistake.

The impression prevails that the Protective Association was gotton up to resist the claims of the International Tooth Crown Company, and for that only. While it is true that the demands of this company was the incentive as the most important present question to be inquired into and determined, those who have inaugurated this movement have broader and more far-reaching ends in view. It is hoped that a fund of large amount can be raised, which shall be safely invested, and produce an income which may be used now and in years to come in any legitimate and proper manner for the benefit of the profession. At present the profession is menaced by what many feel to be unjust claims from patentees. If we use what rightfully belongs to another we should pay for it. It is universally, at this time, regarded as no proof of right that the Patent-Office has granted a patent. It is the universal practice to test in the courts all patents which are found valuable enough, and not until any patent has had a full and fair trial is it considered to be established and valuable.

Fair and honorable men would not enter into combination and raise a fund to enable them to resist a just claim or freely to use what was not theirs. But fair and honorable men may with propriety combine to raise a fund to secure a full and thorough investigation of the justice of any claim, which may now or in the future be made for anything about which there may be doubt. The necessity for this and the justice of it rest upon the fact that no individual has himself enough at stake to justify the expense of testing a claim single-handed or the time and financial ability to do this fully and thoroughly.

We live in communities where each man is dependent for his safety and success upon others. No man can be independent. A member of a community has duties and obligations beyond himself. He should pay his debts and support his family as his first duty, but he cannot be absolved from the moral obligation to give of his means, in proportion to his success, for objects outside of his immediate surroundings. All movements for the good of society should not only enlist his sympathy but be helped by him in more substantial ways. He cannot do his whole duty and be clean through and through if he lends his aid only to those objects in which he has a particular and selfish interest.

If these principles are correct, it makes no difference to any one of us whether we use a method which is to be investigated or not, provided that the question is of importance to others of our co-laborers or to the profession at large. Our duty is to assist in every good movement so far as our means will permit. We cannot shirk our duty and let a few do the work for the good of all and retain our self-respect.

In regard to the Protective Association, if you will carefully read the circular and by-laws I need say no more. It is all fully stated.

The object is mutual assistance and mutual protection,—not of every one, but of the members only. If you, while a non-member, get into trouble, the Association will not help you nor even receive you as a member. You are an outsider and must so remain. But if a member be assailed, the Association will take upon itself the investigation of the justice of the claim against him, and, if it is an open question, will assume all the work and expense of his defence.

The ultimate object of the association is that truth, fair dealing, and justice may prevail, and that wrong, bulldosing, intimidation of the weak, and injustice may be met by an efficient defence and overthrown.

While certain claims assume great prominence at present, no one can foretell what other claims may arise in the near future. If we are to judge by the past, there will be many such claims and of a nature little dreamed of now.

I urge this object upon you now, not because we are face to face with a certain company's claims, but because I solemnly believe that if the present movement fails to enlist the full co-operation of the profession throughout the country, it is extremely doubtful whether it will ever be attempted again. I have perfect confidence in the uprightness, steadfastness, honesty, and grit of the gentleman who is at the head of this movement, who is giving his valuable time, working day and night, and for what? That some stranger, a fellow-member in Boston, Springfield, or Hartford, with little business ability or pecuniary means, can be protected in his rights.

I have confidence that the money of the members is in safe hands, as each member has a receipt from the vice-president of the First National Bank of Chicago, a bank of good reputation.

I have confidence that the management of the association is judicious and wise and that the legal talent is able and honest.

I am happy that there are some who are willing to do the work while I have only to pay my paltry ten dollars.

The question has been put to me, as a personal one, whether I have any right, being a licensee of a certain patent company, to join the Protective Association and urge others to become members.

I have carefully considered this question in its legal and moral aspects, and am satisfied that I have the right legally and morally. To establish this position to your satisfaction I must tell the story at some length. A method of practice is invented and comes into quite general use. I use it, thinking it public property. Years afterwards it is patented, and the patent becomes the property by assignment of a company. The agent of the company visits me and asks me to settle for infringement. While I may have infringed legally, I have not done so morally. I offer to settle with him, but he will do so only on condition that I take out a license for future use of the invention, so worded as to be, to my apprehension, untrue, insulting, and degrading. I decline to take the license, and he threatens me that he will immediately apply to the court for an injunction to issue against me. He offers me one of two alternatives, a license or an injunction, a humiliation or a public injury to my business, and wants an immediate answer. After parleying, he agrees to a modification of the license so as to obviate my objection, and says he will send the modified license to headquarters for approval. In a few days he reappears and reads me a letter from head-quarters saying he is authorized to execute the license as modified by me. I have witness to the reading by the agent of this letter. I then execute the license, pay my money for the license, and for past infringement. The agent then says he must send the license to New York to be countersigned at head-quarters. In a few weeks the manager of the company calls upon me, tells me the agent's act was unauthorized, tenders me back my check and the license I had signed, and presents me a new license with the objectionable features, and threatens me with an injunction if I refuse. I find I am caught in a trap; that I have given myself away, and must either sign or fight the company single-handed, and at a disadvantage from having made returns of cases of the use of the invention. This came also, at a time when, from family sickness and sore distress, I had no time, energy, or leisure for a legal contest. I did what I should do again and always under similar circumstances. I signed the license, pocketed the imposition, and was at peace. There was then no Protective Association. What I did I did under intimidation and falsehood. It was as if, on a lonely road on a dark night, a voice cries, "Money or your life!" and life is saved at any expense. The offer to pay for what one had innocently taken of what was claimed as another's property was insultingly spurned and the threat made to blight one's business reputation by a judicial decision that he was an infringer or a thief. It has been universally declared by our courts that a check or note signed under duress or intimidation was void. The obtaining of this license from me was analogous. There is, notwithstanding any expression in it, no abridgment of my full and perfect legal liberty to assail it as though it had never been signed. Ever since that time my resentment at the deception, falsehood, intimidation, and brow-beating has been increasing until now my whole moral nature rises in rebellion against a company which prefers "ways that are dark and tricks that are vain" instead of fair, square, and honorable dealing.

*Dr. S. G. Stevens.*—If you had at that time gone to head-quarters and made them a tender of legal money enough to cover all their claims against you, would they not have been obliged to receive it in settlement without your taking a license?

*Dr. Shepard.*—I cannot answer positively. I have understood that some have successfully done this, but I do not know it. It would seem to be a fair way of settling for all that was past. It would also seem to be fair for a man or company that has anything to sell to put a price on it, and the party desiring to buy can decide whether the price or terms are satisfactory, and buy or not as may seem judicious. The acceptance of pay for the past should not be contingent upon an agreement to buy for the future. Dr. Crouse has had a personal suit brought against him by this company, damages being set at sixty thousand dollars for conspiring to injure its business.

*Dr. R. R. Andrews.*—Will the money of the Protective Association be used to defend him in this suit?

*Dr. Shepard.*—Not one cent of the Association's money. At Saratoga, last summer, two thousand six hundred dollars was subscribed for this purpose by gentlemen of the profession, and the American Dental Association, by vote, appropriated one thousand dollars for the same purpose. The suit against him is personal and entirely separate from the work of the Protective Association.

*Dr. R. R. Andrews.*—Can you tell us who are members of the Protective Association?

*Dr. Shepard.*—I cannot, and I have understood that it is deemed not desirable that the names or number of members shall be publicly known. Any man can say whether he is a member, if he pleases, but it is considered advisable by the managers of the association that they should keep to themselves the list of members.

It may be of interest to know that the Pope Manufacturing Company have so many patents upon bicycles that they have for years controlled the manufacture throughout the whole country, and that no one could manufacture a bicycle without taking a license from them and paying them a royalty. They have managed the business with a very high hand it is said. One of their licensees, who has paid them about twenty thousand dollars in royalties, resisted their claims and was sued. Mr. Offield, the same gentleman who is the attorney for the Protective Association, defended the case, and got a favorable verdict. The ground of successful defence was that no holder of a patent had a right so to manage the business as to cripple, retard, hamper, or paralyze the business of the country; such a means of managing a patent was declared by the court as contrary to public policy. I have understood that the Pope Company has appealed from this decision. The decision is interesting as showing the drift of patent litigation.

*Dr. Bartholomew.*—I wish to ask Dr. Shepard if he intends to take out a license for the coming year?

*Dr. Shepard.*—I do not expect to do so. I am not yet advised as to whether those who have been licensees occupy any different position, legally, from those who have not. It is my intention to act in harmony with the profession, and, now that we have an association for mutual assistance, to do exactly as my attorney, the counsel of the Protective Association, shall advise. In conclusion, let me urge you all to assist in this movement. There are gentlemen here who have the by-laws for you to sign, and who will be pleased to take your names and money and forward them to Dr. Crouse.

*Dr. Stevens.*—Perhaps there are some here who have never read the circulars of the Dental Protective Association, and I would like to ask Dr. Bartholomew if he will please read the list of patents now held by the Tooth Crown Company?

Dr. Bartholomew read from the circular.

*Dr. A. M. Dudley.*—I understand this Protective Association to be somewhat similar to the Association we formerly had here in New England, when some other patents were being tested. We had then what was known as the New England Dental Association. I was the secretary and treasurer, and I can therefore give some information as to what it will cost one if he attempts to do his own fighting on these cases. Some of you remember that every quarter you would receive a notice from me calling for your dollar, and the average cost to each member was eight dollars. You remember, it was a member of this society whose case was made a test case, and the cost of this case in the Circuit Court was paid by myself. You remember our generous friend, the late S. S. White, assumed the liability of this case. After it had gone through the Circuit Court it was again reopened and begun all over, and was again carried through the Circuit Court and decided against us. It was then carried to the Superior Court, and finally was decided against us, and all the costs and expenses were paid by Dr. White. All other cases pending, when this was tried, were kept on file. In the New England Circuit the average expense for each case was sixty-five dollars. So each member of the New England Association, who had paid in only eight dollars, had their costs paid by the association. Now if one attempts to carry his own case through the court he will have, in addition to the expenses of the court, that of the lawyer, who conducts his case for him. Now whichever way you look at it, it is a good investment to put ten dollars into this Dental Protective Association. For by putting in only ten dollars the whole costs of your suit and the lawyer who defends your case will be met from the funds of the association.

Discussion closed.

